# Transthoracic Lung Ultrasound in Systemic Sclerosis-Associated Interstitial Lung Disease: Capacity to Differentiate Chest Computed-Tomographic Characteristic Patterns

**DOI:** 10.3390/diagnostics15040488

**Published:** 2025-02-17

**Authors:** Cinzia Rotondo, Giuseppe Busto, Valeria Rella, Raffaele Barile, Fabio Cacciapaglia, Marco Fornaro, Florenzo Iannone, Donato Lacedonia, Carla Maria Irene Quarato, Antonello Trotta, Francesco Paolo Cantatore, Addolorata Corrado

**Affiliations:** 1 Rheumatology Unit, Department of Medical and Surgical Sciences, University of Foggia, 71122 Foggia, Italy; 2 Rheumatology Service, Internal Medicine Unit “F. Miulli” General Hospital, Acquaviva delle Fonti, 70021 Bari, Italy; 3 Department of Medicine and Surgery, LUM “G De Gennaro”, Casamassima, 70010 Bari, Italy; 4 Reumatology Unit, Dipartimento di Medicina di Precisione e Rigenerativa e Area Jonica, Università degli Studi di Bari Aldo Moro, 70121 Bari, Italy; 5 Institute of Respiratory Diseases, Department of Medical and Surgical Sciences, University of Foggia, 71122 Foggia, Italy

**Keywords:** interstitial lung disease, usual interstitial pneumonia, non-specific interstitial pneumonia, lung ultrasound (LUS), B-lines

## Abstract

**Background/Objectives**: Even today, interstitial lung disease (ILD) is diagnosed by chest high-resolution computed tomography (lung HR-CT). Large amounts of data are available about the usefulness of transthoracic lung ultrasound (LUS) in ILD. This study aimed to evaluate the transthoracic LUS capacity to discriminate different ILD patterns in systemic sclerosis (SSc) patients, such as usual interstitial pneumonia (UIP), non-specific interstitial pneumonia (NSIP) with ground glass opacification/opacity (GGO), and NSIP with GGO and reticulations, as well as the possibility of identifying progressive fibrosing ILD. **Methods**: We enrolled SSc-patients attending the outpatient Clinic of the Rheumatology Unit of Policlinico of Foggia and the Rheumatology Unit of Policlinico of Bari who satisfied these inclusion criteria: age older than 18 years; the satisfaction of ACR/EULAR 2013 classification criteria for SSc; chest HR-CT scan within three months before or three months after transthoracic LUS evaluation; and availability of recent and complete pulmonary function test. The exclusion criteria were as follows: history or recent reactivation of chronic obstructive pulmonary disease, lung cancer, lung infection, heart failure, pulmonary oedema, pulmonary arterial hypertension, acute respiratory distress syndrome and diffuse alveolar haemorrhage and thoracic surgery. All enrolled SSc-patients underwent transthoracic LUS, performed by an experienced sonographer. The ILD diagnosis and the respective patterns were assessed by chest HR-CT, which still represents the best diagnostic tool. **Results**: ILD was observed in 99 (63.5%) patients. Of these, 25% had the UIP pattern and 75% the NSIP pattern (46 with GGO, 28 with GGO and reticulations). By receiver operating characteristic (ROC) curve analysis, higher values of accuracy, sensitivity, specificity, and negative clinical utility index (CUI) were found for pleural line irregularity (0.84 (95% CI: 0.75–0.91), 96%, and 73.6%, *p* = 0.0001; 0.72), and pleural line thickness (0.84 (95% CI: 0.74–0.91), 72%, and 96.4%, *p* = 0.0001; 0.85) for detecting the UIP pattern. The best performance among transthoracic LUS signs for NSIP with the GGO pattern was observed for B-lines (accuracy: 0.88 (95% CI: 0.80–0.93), sensitivity: 93.4% and specificity: 82.4, *p* = 0.0001; CUI+: 0.75, CUI−: 0.77). LUS signs with higher accuracy, sensitivity, and specificity for NSIP with GGO and reticulations were pleural line irregularity (0.89 (95% CI: 0.80–0.95), 96.4%, and 82.4%, *p* = 0.0001) with CUI−: 0.72, and B-lines (0.89 (95% CI: 0.80–0.95), 96.4%, 82.4%, *p* = 0.0001), with CUI+: 0.80 and CUI−: 0.70. Furthermore, a total number of B-lines > 10 maximises LUS performance with 92.3% sensitivity, and an accuracy of 0.83 (*p* = 0.0001) for detecting the NSIP pattern, particularly GGO. A sample-restricted analysis (66 SSc patients) evidenced the presence of progressive fibrosing ILD in 77% of these patients. By binary regression analysis, the unique LUS sign associated with progressive fibrosing ILD was the presence of pleural line irregularity (OR: 3.6; 95% CI 1.08–11.9; *p* = 0.036). **Conclusions**: Our study demonstrated that transthoracic LUS presented a high capacity to discriminate the different patterns of SSc-ILD. Therefore, the hypothesis that transthoracic LUS is an effective screening method for the evaluation of the presence of SSc-ILD and establishing the correct timing of chest HR-CT, in order to avoid patients receiving excessive exposure to ionising radiation, is supported.

## 1. Introduction

Interstitial lung disease (ILD) is one of the most frequent clinical features in systemic sclerosis (SSc), estimated in 53% of patients with SSc with a diffuse cutaneous subset and in 35% of patients with a limited cutaneous subset [1]. Together with pulmonary arterial hypertension, despite advances in therapeutic management, it currently represents the most frequent cause of death in patients with SSc (ranging between 17% and 35%) [2,3,4,5,6].

The American College of Rheumatology (ACR) and the American College of Chest Physicians (CHEST) Guideline have recently confirmed that the best instrumental method for diagnosing and following ILD in rheumatic disorders, including SSc, is chest high-resolution computed tomography (chest HR-CT) [7], due to its capacity to recognise SSc pulmonary disease [8,9] and to assess the ILD extension [10,11,12]. Given the subclinical onset and the need to diagnose SSc-ILD as early as possible [13], in order to try to slow down its progression through pharmacological therapy [14], a pulmonary function test (PFT) every 3–6 months in the first year of diagnosis, and then less frequently once stable, and lung HR-CT, when necessary on the base of reported symptoms, clinical examination, and PFT, are recommended to be performed, especially for the population considered to be at the highest risk (anti–Scl-70 positivity, antinuclear antibody with nucleolar pattern, diffuse cutaneous subtype, male sex, African American race, early disease (first 5–7 y after onset), and elevated acute phase reactants) [7].

The most frequent (70–78%) lung CT pattern of ILD in SSc, above all in early phases of the disease, is non-specific interstitial pneumonia (NSIP) characterised by the presence of ground glass opacities (GGO) and reticulations, and less commonly (10–13.8%) by usual interstitial pneumonia (UIP), identified by honeycombing and traction bronchiectasis [15,16].

In the last decade, however, the issues of high cost and the risk related to excessive exposure to ionising radiation, which every CT scan induces, as well as the increased risk of developing neoplasms (especially breast carcinomas) in patients with SSc [17], have induced the scientific community to search for new instrumental methods that are less harmful to the patient. Among these, transthoracic lung ultrasound (LUS) has assumed considerable importance, due to preliminary data supporting a positive association between LUS signs and CT findings [18,19,20,21,22,23,24,25,26], but no study has ever compared specific tomographic patterns of ILD with particular signs of transthoracic LUS in SSc patients.

This study aimed to evaluate the LUS capacity to discriminate different ILD patterns, such as UIP, NSIP with GGO, and NSIP with GGO and reticulations, in SSc patients. Furthermore, we wanted to evaluate the presence of any characteristic LUS signs to identify progressive fibrosing ILD.

## 2. Materials and Methods

We performed a cross-sectional study, conducted from January 2023 to November 2024. The enrolled patients were followed at the outpatient clinic of the Rheumatology Unit of Policlinico of Foggia and the Rheumatology Unit of Policlinico of Bari. All the patients satisfied the ACR/EULAR 2013 classification criteria for SSc [27]. The clinical examination at enrolled visit included age, disease history, medications, visceral involvement, and laboratory test for reactants of the acute phase of inflammation (erythrocyte sedimentation rate, C-reactive protein), antinuclear antibodies (assessed by immunofluorescence) and extractable nuclear antigens antibodies (evaluated by immunoblotting), the presence of ILD and its pattern at chest HR-CT, the respiratory capacity by pulmonary function test, evaluation of the presence of main LUS signs of ILD, and the assessment of nailfold capillaroscopy patterns. The extent and severity of skin thickening were evaluated by the modified Rodnan skin score.

The inclusion criteria were as follows: age older than 18 years, the satisfaction of ACR/EULAR 2013 classification criteria for SSC [27]; chest HR-CT scan within three months before or three months after transthoracic LUS evaluation; and availability of recent and complete pulmonary function test.

The exclusion criteria were as follows: history or recent reactivation of chronic obstructive pulmonary disease, lung cancer, lung infection, heart failure, pulmonary oedema, pulmonary arterial hypertension, acute respiratory distress syndrome, and diffuse alveolar haemorrhage and thoracic surgery.

### 2.1. Chest High-Resolution Computed Tomography

Chest HR-TC evaluation was performed with a high-resolution spiral technique at the patients’ reference centres, using a multi-detector CT scanner with 64 channels (Toshiba, Tokyo, Japan). The standard parameters for CT acquisition were as follows: tube voltage, 120 kVp; tube current, standard (reference mAs, 60–120); slice thickness, 0.5 mm; and reconstruction interval, 0.5–1.0 mm. All chest HR-CT images were captured at full inspiration, with the patient in the supine position and without contrast medium. Each exam, recorded in digital format, was acquired and subsequently examined by the same operator. The lung field was divided into three segments (basal, middle and apical), which did not correspond to the lung lobes. The inferior angle of the scapula was considered a landmark for the subdivision between basal and middle segments, while the middle and apical segments were divided to be constituted by the same number of intercostal spaces. The segmentation was used to standardize the subdivision of transthoracic LUS areas and tomographic lung segments. For each lung field, the examiner assessed the presence of characteristic lesions (covering at least 20% of the segment area) for NSIP with GGO only, NSIP with GGO and reticulations, and UIP [28]. In patients with ILD, the lung segments characterised by the presence of lesions were considered and compared with the transthoracic LUS findings.

The presence of progressive fibrosing ILD was defined, as suggested by Raghu G et al. [29], by worsening of respiratory symptoms, the evidence of radiographic progression, and/or absolute changes in forced vital capacity (FVC) or diffusing capacity of the lung for carbon monoxide (DLCO) at the enrolled visit compared to those performed by the patient in the previous year (only in patients who had such data available).

### 2.2. Transthoracic Lung Ultrasound

Transthoracic lung ultrasound (LUS) was assessed according to the principal recommendation [30], evaluating all scanning lung fields on the anterolateral (28 sites) and the posterior thorax (30 sites). All transthoracic LUS were performed by a blinded certified operator at the University Hospital of Foggia. The examination was conducted with the patient sitting and their hands resting on their knees for posterior scanning, while the patient adopted a supine position for anterolateral acquisitions. The transthoracic LUS was performed, by an expert sonographer, with a Philips Epiq 7 ultrasound system (Philips Ultrasound; Bothell, WA, USA), using a convex probe (3.5–5 MHz) or a linear transducer (8.0–12.0 MHz), where appropriate, to improve the identification of pleural alterations.

**Figure 1:**
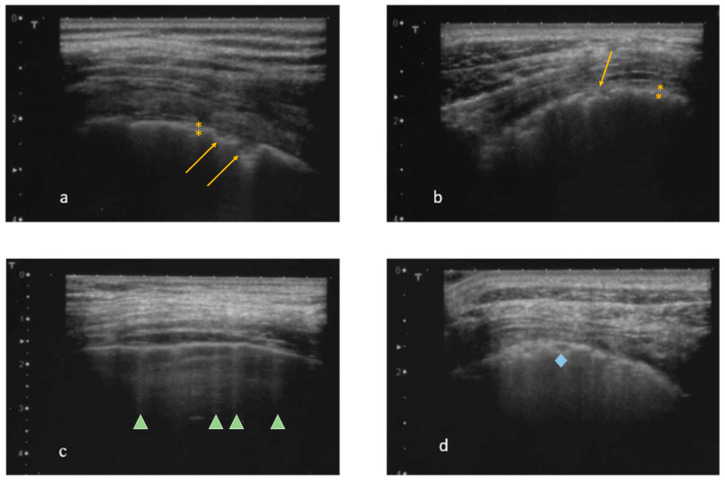
Echograms: signs detected by transthoracic lung ultrasound (linear probe between 7 and 10 MHz); (**a**) thickening (yellow asterisk) and irregularity (yellow arrow) of the pleural line; (**b**) thickening (yellow asterisk) and irregularity (yellow arrow) of the pleural line; (**c**) B-lines (green arrowheads); (**d**) subpleural cysts (blue rhombus).

In each intercostal space examined, the following ultrasonographic characteristics of the pleural line were evaluated:

- Thickness of the pleural line, abnormal if >2.8 mm (Figure 1a,b) [31];
- Irregularities of the pleural line, abnormal if extended more than 3 mm (Figure 1a,b) [31,32];
- Mobility of the pleural line, in particular, the presence of sliding signs;
- The presence and total number of B-lines (abnormal in the presence of 3 or more B-lines in at least two consecutive scans or the presence of at least 5 B-lines in the entire intercostal space examined) (Figure 1c) [31,33]. The total number of B-lines was given by the sum of all the B-lines found in the different fields examined;
- The presence of at least one subpleural cyst (Figure 1d), defined as hypo-echoic lesions that interrupt the pleural line;
- The presence of the pleural effusion.

### 2.3. Pulmonary Function Test

Forced expiratory volume in 1 s (FEV1) and FVC were measured with standard spirometry Sensormedics (Milano, Italia). The PFT was conducted with three inspiratory and expiratory manoeuvres and was considered reliable when the values obtained were repeatable. The values were expressed as the percentage of predicted, using the Quanjer and Stocks equations.

DLCO was assessed with the “single breath” technique and adjusted for haemoglobin and carbon monoxide (CO) levels. The results were recorded as percentages of predicted values.

### 2.4. Statistical Analysis

The results were expressed as means (m) ± standard deviation (sd); categorical variables were expressed as numbers (percentage). The normality of the distribution of the study variables was verified with the Kolmogorov–Smirnov test.

Statistical differences between the two groups were analyzed with the t-student test for unpaired data. Multiple comparisons of continuous variables were analyzed with analysis of variance (ANOVA) followed by the Bonferroni test.

Pearson χ2 and Fisher’s exact test, followed by Z-test, were used to compare categorical variables and percentages. Pearson Correlation was used to evaluate the correlation between the number of B-lines and the Warrick score.

The ROC curve was used to compare the diagnostic performance of each transthoracic LUS sign with chest HR-CT patterns of ILD as NSIP with GGO, NSIP with GGO and reticulations, and UIP, assessing the area under the curve (AUC). The AUC values of 0.50–0.59, 0.60–0.69, 0.70–0.79, and ≥0.80 were defined as none, poor, acceptable, and excellent accuracy, respectively [34].

The optimal cutoff for the number of B-lines in the patterns NSIP with GGO, and NSIP with GGO and reticulations, corresponding to the maximum sum of sensitivity and specificity, were computed by ROC curve.

Positive and negative clinical utility index (CUI) values were calculated as the product between sensitivity and positive predictive value (PPV), and the product between specificity and negative predictive value (NPV), respectively. Grading was performed using the categories excellent utility ≥ 0.81, good utility ≥ 0.64 and fair utility ≥ 0.49 and poor utility < 0.49, as suggested by Mitchell [35].

Binary logistic regression was used to evaluate transthoracic LUS signs (independent variables) that were associated with progressive fibrosing ILD (dependent variable).

Statistical significance was defined as a value of *p* ≤ 0.05.

Statistical analysis was performed with IBM SPSS Statistics 26.

### 2.5. Ethics Approval

Our study was approved by the local Ethics Committee (115/C.E.—21 September 2021). All participants gave their informed consent to participate in this study.

## 3. Results

One hundred fifty-six patients were consecutively recruited for this study. All patients had SSc (mean age 59.0 ± 12.4 years; and mean disease duration 32 ± 10.8 years) and satisfied the inclusion criteria. ILD at chest HR-CT was detected in 99 (63.5%) studied patients, of whom 25 (25%) had the UIP pattern, and 74 (75%) the NSIP pattern (46 with GGO alone, and 28 with GGO and reticulations).

A comparison of the main demographic and clinical characteristics of the study groups is shown in Table 1. A higher presence of anti-Scl-70 antibodies was found in patients with ILD (any CT patterns) compared with No-ILD patients. Lower values of FVC in pulmonary function tests were detected in the UIP (84.8 ± 18.3), and NSIP with GGO and reticulations (84 ± 19.4) groups. Regarding other specific CT lung abnormalities, a higher rate of micro-nodules was found in the NSIP with GGO group (56.5%). More extensive and severe ILD, as assessed by the Warrick score, was recognised in the UIP (10.2 ± 4) and NSIP with GGO and reticulations (10.8 ± 1.7) groups (Table 1). A positive correlation between the Warrick score and the number of B-lines was found (r^2^: 0.59; *p* = 0.0001).

### 3.1. LUS Signs and UIP Chest CT Pattern

Regarding UIP at chest CT, the rate of presence of pleural line irregularity (96% vs. 26%), sliding sign (20% vs. 0%), pleural line thickness (72% vs. 3.5%), B-lines (56% vs. 17.5%), and subpleural cysts (58% vs. 9%) were higher compared to the No-ILD group of patients (Table 1). Furthermore, the frequency of the presence of pleural thickness was higher in the UIP pattern than in NSIP with GGO (72% vs. 36%) (Table 1).

**Figure 2:**
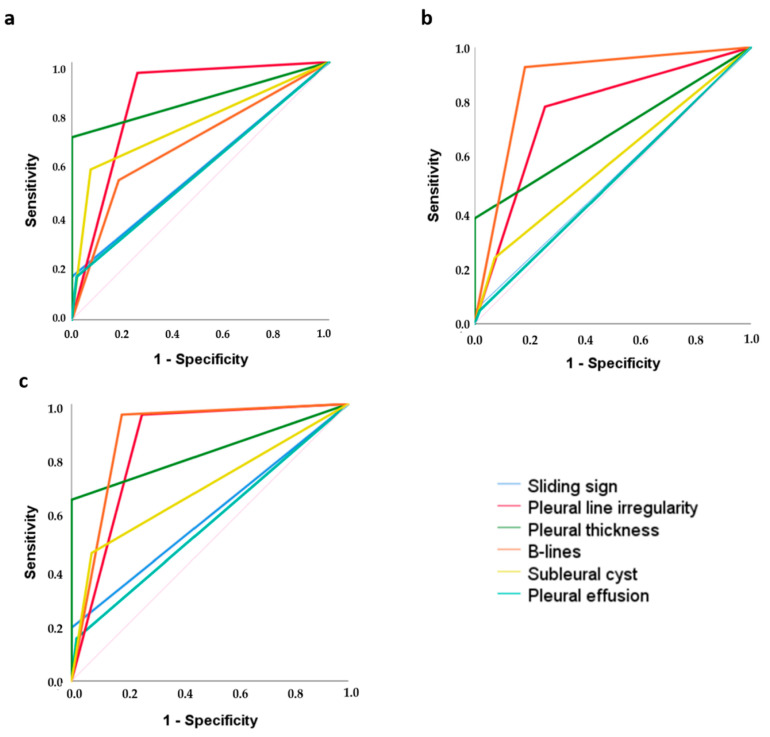
Graphical representation of ROC curve analysis of different transthoracic LUS signs for detecting (**a**) UIP CT ILD pattern; (**b**) NSIP with GGO CT ILD pattern; (**c**) NSIP with GGO and reticulations CT ILD pattern.

In the ROC curve analysis of the UIP pattern, better performance was found in terms of sensitivity and sensibility, with excellent accuracy and CUI positive and negative values, for pleural line irregularity (with a higher negative predictive value), and pleural line thickness (Table 2, Figure 2a). Poor CUI positive values were found for B-lines and pleural effusion. The subpleural cyst assessment had good accuracy, specificity and CUI negative values, but a low value of sensitivity (Table 2).

### 3.2. LUS Signs and NSIP with Ground Glass Lung CT Pattern

Concerning the NSIP with GGO pattern, the transthoracic LUS signs observed with higher frequency were pleural line irregularity (76% vs. 26%) (but significantly lesser than for the UIP pattern), B-lines (93.5% vs. 17.5%), compared with the No-ILD patients. In particular, there was a significant difference in the rate of the presence of B-lines (93.5% vs. 56%) and the total number of B-lines (69.5 ± 10.7 vs. 29 ± 10.2; *p* = 0.0001) between the NSIP with GGO pattern and the UIP pattern (Table 1).

By ROC curve analysis, the best performance among transthoracic LUS signs for the NSIP with GGO pattern was observed just for B-lines, with excellent accuracy, good CUI positive and negative values, high sensitivity, and a high negative predictive value (Table 2, Figure 2b). Regarding the cut-off of total B-line numbers useful for discriminating the presence of NSIP with GGO pattern, a total number of B-lines > 10 maximises the transthoracic LUS performance, with 92.8% sensitivity, 96.4% specificity, and an accuracy of 0.945 (*p* = 0.0001). Low values of sensitivity, negative predictive value, and acceptable and poor accuracy were found for pleural thickness and pleural line irregularities, respectively (Table 2).

#### 3.2.1. Transthoracic LUS Signs and NSIP with Ground Glass and Reticulations Chest CT Pattern

The NSIP with GGO and reticulations pattern was detected in 28 patients. The principal transthoracic LUS signs assessed with a higher rate in patients with this pattern compared to No-ILD SSc patients were sliding sign (18.5% vs. 0%), pleural line irregularity (96% vs. 26%), pleural line thickness (64% vs. 3.5), B-lines (96% vs. 17.5%), and subpleural cysts (48% vs. 9%). In particular, the presence of B-lines and the number of B-lines were found to be statistically significant between the NSIP with GGO and reticulations group and UIP group, with values of 96% vs. 56%, and 56.6 ± 11.8 vs. 29 ± 10.2, respectively (Table 1).

By ROC curve analysis, the transthoracic LUS signs with higher accuracy, sensitivity, specificity and CUI for NSIP with GGO and reticulations were firstly B-lines, followed by pleural thickness and pleural irregularity, which presented excellent and good CUI negative values, respectively. Concerning the cut-off number of B-lines, the presence of >10 B-lines maintains good sensitivity (91.3%) for the NSIP pattern with GGO and reticulations, but loses some points in terms of specificity (96.4%) and accuracy (0.934; *p* = 0.0001) compared to the NSIP pattern with GGO.

The true positive, false positive, true negative and false negative of transthoracic LUS signs for each tomographic ILD pattern are reported in Supplementary Table S1.

#### 3.2.2. Transthoracic LUS Signs and NSIP with Ground Glass and Reticulations Lung CT Pattern

In 66 patients, it was possible to perform a comparative evaluation to establish the presence of progressive fibrosing ILD, and 77% of these patients presented the criteria for progressive fibrosing ILD. A higher rate of pleural line irregularities was found in the group of patients with progressive fibrosing ILD compared to those with ILD (71% vs. 40%, *p* = 0.033). No other significant differences in transthoracic LUS signs were found between the group of patients with ILD and those with progressive fibrosing ILD. A comparison of the principal clinical and instrumental characteristics between the patients with progressive fibrosing ILD and those with non-progressive ILD are shown in Table 3.

By binary regression analysis, the unique transthoracic LUS sign associated significantly with progressive fibrosing ILD was the presence of pleural line irregularity (OR: 3.6; 95% CI 1.08–11.9; *p* = 0.036). No significance was observed regarding the presence or absence of B-lines and their number.

## 4. Discussion

This study evidenced principally that the presence of B-lines at transthoracic LUS characterised the chest HR-CT pattern of NSIP with GGO with excellent accuracy (0.88), sensitivity (93.4%) and negative predictive value (94%), as well as a good CUI negative value (0.775) and good CUI positive value (0.758), with a sensitivity of 82.4%. Furthermore, the best indicators of the UIP tomographic pattern were the presence of pleural line thickness (excellent accuracy (0.842) and CUI negative value (0.856), and good CUI positive value (0.648)), and the presence of pleural line irregularities (good accuracy (0.848) and CUI negative value (0.72)). NSIP with GGO and reticulations was characterised in transthoracic LUS principally by B-lines (with good (>0.64) CUI positive and negative values, excellent accuracy (0.894), sensitivity (96.4%), and a high (97.9%) negative predictive value), and by pleural line irregularities (good CUI negative value (0.816), excellent accuracy (0.851), and a high (96.4%) sensitivity). Pleural line thickness presented, for NSIP with the GGO and reticulations pattern, high specificity (96.4%), accuracy (0.804), a positive predictive value (90%) and an excellent (0.816) CUI negative value.

A large number of studies are available on the use of transthoracic LUS in ILD, although a consensus has never been reached on its real clinical value, the coding and scoring system [14]. In SSc, due to the characteristic and orderly progressive involvement of the lung parenchyma, starting from the subpleural basal–posterior lung interstitium, which is easily viewable by LUS [36], the ultrasound examination, in particular the detection of pleural thickness, has been previously proposed as a timely guide for the execution of chest HR-CT [33,37,38,39]. Our study adds to the other published works, introducing considerations regarding how LUS can differentiate specific lung HR-CT patterns such as NSIP and UIP. Indeed, the present study highlights that transthoracic LUS is a highly sensitive and specific technique in evaluating the presence of typical SSc-ILD tomographic patterns and is also accurate in discriminating its different and characteristic phases. The finding of B-lines on LUS was found to be significantly associated with the presence of active inflammatory phases of SSc-ILD, characterised by GGO, as observed in the NSIP pattern [40,41,42,43]. Irregularities of the pleural line and the thickness of the pleural line are, instead, significantly associated with the presence of honeycombing and traction bronchiectasis, as in the UIP pattern, and therefore, of the fibrosis phase. Therefore, principally, the presence or the absence of B-lines significantly differentiates the NSIP pattern from the UIP pattern.

The presence of transthoracic LUS signs, such as the presence of irregularities of the pleural line, thickness of the pleural line, the presence of subpleural cysts, reduced mobility of the pleural line and the presence of B-lines, in patients with pulmonary fibrosis was first described in 1997 [44]. Other studies have evaluated the presence of these same transthoracic LUS signs in ILD secondary to sarcoidosis, rheumatoid arthritis, SSc, mixed connective tissue disease, Sjogren’s syndrome and primary biliary cirrhosis, demonstrating their presence also in these secondary forms of ILD [31], and highlighting, in particular, that the presence of B-lines distributed throughout the lung area is associated with more severe pulmonary fibrosis [45]. Concerning the transthoracic LUS evaluation of ILD in connective tissue diseases, in previous studies, it has been observed that a greater number of B-lines could identify SSc-ILD [31,45,46]; and, in particular, that the number of B-lines correlates with the tomographic Warrick score, an index of severity of SSc-ILD [19]. Furthermore, the high sensitivity of B-lines in evaluating the presence of SSc-ILD has been described both in patients with a diagnosis of very early SSc [47] and those with a diagnosis of SSc [33].

The high frequency and sensitivity found in the present study between the presence of B-lines and the tomographic pattern of NSIP with GGO, characteristic of active inflammatory processes [48], could be attributed to the presence of inflammatory infiltrate that would result in variation in the acoustic impedance of the lung parenchyma, creating the air–water interface necessary for the generation of B-lines, as demonstrated in previous studies [49,50,51].

Concerning the cut-off of B-lines, the limit of >10 B-lines found to be effective for recognising ILD confirms what was previously found for ILD-SSc [52,53]. However, in our study, we found a high sensitivity for the NSIP pattern, especially for the GGO-only pattern, but also for the GGO and reticulations pattern. These findings have not been specifically evaluated in previous studies. However, few data support the presence of B-lines in both GGO and honeycombing patterns [14,37]. Still, no study has ever investigated the possible association between specific chest HR-CT patterns, such as UIP and NSIP, and transthoracic LUS signs. These results could indicate a greater sensitivity for the more inflammatory phases, which would result from the presence of a greater inflammatory infiltrate with a subsequent increase in acoustic impedance.

In a previous study, a low specificity (55%) of B-lines was highlighted for SSc-ILD, despite a high sensitivity (100%); this finding was attributed to the probable presence of pulmonary oedema [31]. Our study improved the previous data with higher sensitivity (93.4% and 96.4%), and higher specificity (82.4% and 82.4%) for both NSIP with GGO and NSIP with GGO and reticulations, respectively, with good positive and negative CUI values.

Regarding the tomographic pattern of UIP, a high sensitivity of transthoracic LUS signs was observed, such as the thickness of the pleural line, the presence of irregularities of the pleural line and the presence of subpleural cysts. This finding could be due to the presence of structural subversion of the lung parenchyma (complete loss of the typical acinar structure of the lung) observed in cases of stabilized pulmonary fibrosis, characterised tomographically by the presence of honeycombing [54].

No data are available on the use of transthoracic LUS in progressive fibrosing ILD; for the first time in this study, we observed a strict association between this ILD pattern and the presence of pleural line irregularities. Probably, the subversion of the parenchymal structure could explain the irregularities of the pleural line visible with transthoracic LUS. Observational studies with larger patient samples would serve to confirm this finding.

In the group of patients enrolled in this study, the percentage of SSc-ILD (63%) is in line with values reported in other studies [1,55]; and the ENA specificity found most frequently in these patients is anti-Scl-70, as observed previously [56].

Therefore, transthoracic LUS, due to its versatility, ease of handling and low maintenance costs, could assume considerable clinical importance for evaluating SSc-ILD. Furthermore, the ease of interpretation of transthoracic LUS images and the rapid execution times (minimum duration < 10 min [33] and maximum 23 min [53]) make transthoracic LUS an efficient instrumental method from a clinical point of view. 

The accuracy and completeness of the information provided by the tomographic images, currently not obtainable by other methods, still make the chest HR-CT the better instrumental technique for diagnosing SSc-ILD. Although transthoracic LUS will probably not replace the chest HR-CT for the diagnosis of SSc-ILD, at least in the short term, it could be considered a guide to establish the most appropriate timing to perform the chest HR-CT, minimising the risk of exposure to ionising radiation [33]. In recent decades, this latter risk and the need to contain the costs related to the diagnosis and clinical management of patients has led the scientific community to identify new imaging techniques and new predictors of disease [18,19,20,21,22,23,24,25,26,57,58,59,60,61,62]. A transthoracic LUS score with a weighted score for each transthoracic LUS sign would be desirable to improve the diagnostic ability of transthoracic LUS for different HR-CT patterns of ILD. The clinical utility of recognizing the different patterns of ILD-SSc early, and without excessive exposure to ionising radiation, could have important implications in the therapeutic management of patients with SSc. With the advent of artificial intelligence software, it will probably be possible to design multifactorial scores that will meet the needs of clinicians.

Furthermore, considering the good sensitivity values, negative predictive value and negative CUI of the B-lines (for both NSIP patterns) and of the pleural line irregularities (for the UIP and NSIP patterns with GGO and cross-linking), our results strengthen the data on LUS as a promising screening technique for ILD in SSc and as a potential diagnostic tool, significantly impacting the clinical management of SSc patients [63].

There are some limitations to our study. Firstly, the distance between two consecutive B-lines was not reported. This parameter has been shown to correlate with the Warrick score in patients diagnosed with pulmonary fibrosis [64], but its evaluation was not considered useful for the purposes of our study. It would have been, furthermore, interesting to compare the ultrasound data of patients with SSc-ILD with the data of healthy subjects and other groups of patients with pathologies complicated with ILD. However, in other studies conducted on this topic, the same transthoracic LUS signs highlighted in this study were also found for other forms of ILD [31,42,65]. We did not use the scoring system of transthoracic LUS, as it has not yet been validated. Regarding progressive fibrosing ILD, it would have been useful to evaluate the transthoracic LUS in an observational study at two time points in conjunction with the lung HR-CT. Instead, we only have one transthoracic LUS detection in conjunction with the last lung HR-CT performed.

Considering the generalizability of our results, it is important to underline that transthoracic LUS could represent a screening technique, and probably in the future a valid diagnostic tool, for ILD characterised by lesions located in subpleural areas. However, it would be less reliable in ILD characterised by lesions localised along the peribronchiolar, peribronchio–vascular and perilymphatic regions, as in sarcoidosis, bronchiolitis-associated interstitial lung disease, and lymphoid interstitial pneumonia.

## 5. Conclusions

The present study, comparing two instrumental methods (transthoracic LUS and lung HR-CT) demonstrates that there is a good association between these two methods in evaluating the presence of specific patterns of ILD. In particular, transthoracic LUS has shown high sensitivity in distinguishing different tomographic ILD patterns, such as UIP with pleural line irregularities, pleural line thickness and the presence of subpleural cells; NSIP with GGO, characterised by B-lines; and NSIP with GGO and reticulations, characterised by pleural thickness, pleural irregularities and B-lines. Therefore, the hypothesis that transthoracic LUS is an effective screening method for the evaluation of the presence of SSc-ILD and can be used to establish, therefore, the correct timing of a chest HR-CT, in order to avoid patients from excessive exposure to ionising radiation, is supported. Our results also could open new avenues to the possibility of using LUS as a future recognised diagnostic technique in ILD-SSc. Observational studies should be performed to support our results and confirm LUS findings to further characterise the pattern of progressive fibrosing ILD.

## Figures and Tables

**Table 1:** Comparison among study groups of the main demographic, clinical and instrumental characteristics.

	No-ILD	UIP	NSIP with GGO	NSIP with GGO and Reticulations	*p*-Value
*n* (%)	57 (36.5)	25 (16)	46 (29.5)	28 (18)	
F/M *n* (%)	55 (96)/2 (3)	23 (92)/2 (8)	43 (93)/3 (6)	27 (96)/1 (4)	0.788
Age (years) (m ± sd)	57.5 ± 14.4	60.4 ± 11.0	58.2 ± 11.5	62.2 ± 10.1	0.370
Disease duration (years) (m ± sd)	9.7 ± 6.9	10.9 ± 5.9	7.4 ± 5.9	7.8 ± 7.7	0.479
BMI (m ± sd)	25.9 ± 4.1	25.2 ± 6.1	25.2 ± 4.3	26.1 ± 4.5	0.170
ESR (mm/h) (n.r. 0–30) (m ± sd)	19.3 ± 14.8	17.6 ± 14	17.7 ± 13.1	24.5 ± 14.4	0.242
CRP (mg/L) (n.r. 0–10) (m ± sd)	4.8 ± 4.8	5.0 ± 1.1	7.5 ± 1.3	9.6 ± 1.8	0.238
ENA					
Anti CENP-B positivity *n* (%)	42 (74)	3 (12)	6 (13)	6 (23)	0.0001 ^
Anti Scl-70 positivity *n* (%)	10 (17.5)	19 (76)	36 (78)	17 (65)	0.0001 ″
Nailfold Videocapillaroscopy					
Early scleroderma pattern *n* (%)	16 (32)	4 (20)	16 (40)	4 (17)	0.440
Active scleroderma pattern *n* (%)	24 (45)	8 (40)	15 (37)	12 (52)	0.786
Late scleroderma pattern *n* (%)	13 (24)	8 (40)	9 (22.5)	7 (30)	0.443
Cutaneous subsets					
Limited *n* (%)	49 (86)	20 (80)	34 (74)	25 (89)	0.292
Diffuse *n* (%)	8 (14)	5 (20)	12 (26)	3 (11)	0.296
Presence of LUS abnormalities					
Sliding sign *n* (%)	0 (0)	5 (20)	2 (4)	5 (18.5)	0.002 ^§^
Pleural line irregularity *n* (%)	15 (26)	24 (96)	35 (76)	27 (96)	0.0001 ^
Pleural thickness *n* (%)	2 (3.5)	18 (72)	16 (36)	18 (64)	0.0001 °
B-lines *n* (%)	10 (17.5)	14 (56)	43 (93.5)	27 (96)	0.0001 #
B-lines number (m ± sd)	3.5 ± 16.1	29.0 ± 10.2	69.5 ± 10.7	56.6 ± 11.8	0.0001 #
Subpleural cystis *n* (%)	5 (9)	14 (58)	10 (24)	13 (48)	0.0001 ^@^
Pleural effusion *n* (%)	2 (3.5)	4 (16)	2 (4)	4 (14)	0.103
PFT					
FVC% (m ± sd)	108.1 ± 14.4	84.8 ± 18.3	99.5 ± 15.4	84 ± 19.4	0.0001 ^&^
FEV1% (m ± sd)	100.8 ± 22.2	89.5 ± 26.1	90.9 ± 18.3	80.6 ± 19.6	0.010 ^$^
DLCO% (m ± sd)	77.1 ± 16.9	77.2 ± 17	77.7 ± 18	69.1 ± 18.5	0.627
TLC% (m ± sd)	101.6 ± 12.9	75.2 ± 11	92.9 ± 16.2	91.8 ± 33	0.059
RV% (m ± sd)	105.8 ± 24.9	75.6 ± 13.3	94.8 ± 19.0	93.2 ± 15.7	0.171
6MWT (meter) (m ± sd)	475.9 ± 140.8	390.8 ± 132.6	440.3 ± 102.3	366.1 ± 156.7	0.109
Other lung CT abnormalities					
Micro nodules *n* (%)	6 (13)	8 (32)	26 (56.5)	9 (32)	0.0001 *
Warrick Score *n* (%)	0.0 ± 0.0	10.2 ± 4.0	8 ± 3.4	10.8 ± 1.7	0.0001 ^$^

6MWT: 6 min walking test; BMI: body mass index; CRP: C-reactive proteins; CT: computed tomography; DLCO: diffusing capacity of the lungs for carbon monoxide; ENA: extractable nuclear antigen; ESR: erythrocyte sedimentation rate; F: female; FEV1: forced expiratory volume in the first second; FVC: forced vital capacity; GGO: ground glass; ILD: interstitial lung disease; LUS: lung ultrasound; M: male; n.r.: normal range; NSIP: non-specific interstitial pneumonia; PFT: pulmonary function tests; RV: residual volume; TLC: total lung capacity; UIP: usual interstitial pneumonia. * NSIP with GGO vs. UIP *p* ≤ 0.05; NSIP with GGO vs. No-ILD *p* ≤ 0.05; NSIP with GGO and reticulations vs. No-ILD *p* ≤ 0.05; UIP vs. No-ILD *p* ≤ 0.05. ^§^ UIP vs. No-ILD *p* ≤ 0.05; NSIP with GGO and reticulations vs. No-ILD *p* ≤ 0.05. ^ NSIP with GGO vs. No-ILD *p* ≤ 0.05; UIP vs. No-ILD *p* ≤ 0.05; NSIP with GGO and reticulations vs. No-ILD *p* ≤ 0.05. ″ No-ILD vs. NSIP with GGO *p* ≤ 0.05; No-ILD vs. UIP *p* ≤ 0.05; No-ILD vs. NSIP with GGO and reticulations *p* ≤ 0.05. ° UIP vs. NSIP with GGO *p* ≤ 0.05; NSIP with GGO vs. No-ILD *p* ≤ 0.05; NSIP with GGO and reticulations vs. No-ILD *p* ≤ 0.05; UIP vs. No-ILD *p* ≤ 0.05. # UIP vs. No-ILD *p* ≤ 0.05; NSIP with GGO vs. No-ILD *p* ≤ 0.05; NSIP with GGO and reticulations vs. No-ILD *p* ≤ 0.05; NSIP with GGO vs. UIP *p* ≤ 0.05; NSIP with GGO and reticulations vs. UIP *p* ≤ 0.05. ^@^ UIP vs. No-ILD *p* ≤ 0.05; UIP vs. NSIP with GGO *p* ≤ 0.05; NSIP with GGO and reticulations vs. No-ILD *p* ≤ 0.05. ^&^ UIP vs. No-ILD *p* ≤ 0.05; NSIP with GGO vs. No-ILD *p* ≤ 0.05; NSIP with GGO and reticulations vs. No-ILD *p* ≤ 0.05; UIP vs. NSIP with GGO *p* ≤ 0.05; NSIP with GGO and reticulations vs. GGO *p* ≤ 0.05. ^$^ UIP vs. No-ILD *p* ≤ 0.05; NSIP with GGO vs. No-ILD *p* ≤ 0.05; NSIP with GGO and reticulations vs. No-ILD *p* ≤ 0.05; NSIP with GGO vs. NSIP with GGO and reticulations *p* ≤ 0.05.

**Table 2:** ROC curve analysis. Sensitivity, specificity, accuracy, positive predictive value, negative predictive value, and clinical utility index values (positive and negative) of different lung ultrasound signs for detecting specific ILD CT lung patterns (UIP, NSIP with GGO, NSIP with GGO and reticulations).

	Sensibility % (95% CI)	Specificity % (95% CI)	PPV %	NPV %	CUI +	CUI −	Accuracy (95% CI)	*p*-Value
UIP								
Sliding sign	20 (6.8–100)	100 (93–100)	100	74	0.2	0.74	0.60 (0.48–0.70)	0.01
Pleural line irregularity	96 (79.6–99.6)	73.6 (60.3–84.5)	61.5	97.7	0.591	0.72	0.848 (0.75–0.91)	<0.0001
Pleural thickness	72 (50.6–87.9)	96.4 (87.9–91.6)	90	88.7	0.648	0.856	0.842 (0.74–0.91)	<0.0001
B-lines	56 (34.9–75.6)	82.4 (70.1–91.3)	58.3	81	0.327	0.668	0.692 (0.58–0.79)	0.007
Subpleural cystis	58.3 (36.6–77.9)	91.2 (80.7–97.1)	73.7	82.5	0.413	0.753	0.748 (0.63–0.83)	<0.0001
Pleural effusion	16 ((4.5–36.1)	96.4 (87.9–91.6)	66.7	72.4	0.107	0.698	0.562 (0.448–0.67)	0.110
NSIP with GGO								
Sliding sign	4.35 (0.5–14.8)	100 (93.5–100)	100	56.4	0.043	0.564	0.552 (0.42–0.62)	0.152
Pleural line irregularity	76.9 (61.2–87.4)	73.6 (60.3–84.5)	71.2	82.4	0.572	0.607	0.749 (0.654–0.829)	<0.0001
Pleural thickness	35.5 (21.9–51.2)	96.4 (87.9–99.6)	88.9	64.7	0.309	0.624	0.660 (0.56–0.75)	<0.0001
B-lines	93.4 (82.1–96.8)	82.4 (70.1–91.3)	81.8	94	0.758	0.775	0.88 (0.80–0.93)	<0.0001
Subpleural cystis	23.1 (12.1–39.5)	91.2 (80.7–97.1)	66.6	59.1	0.145	0.539	0.575 (0.47–0.67)	0.0494
Pleural effusion	4.44 (0.0–7.9)	100 (93.7–100)	3.9	4.3	0.004	0.002	0.505 (0.40–0.60)	0.813
NSIP with GGO and reticulations								
Sliding sign	19.5 (6.3–38.1)	100 (93.5–100)	100	71.3	0.179	0.713	0.593 (0.47–0.7)	0.015
Pleural line irregularity	96.4 (81.7–99.9)	73.6 (60.3–84.5)	64.3	97.7	0.620	0.720	0.851 (0.75–0.91)	<0.0001
Pleural thickness	64.2 (44.1–81.4)	96.4 (87.9–99.6)	90	84.6	0.579	0.816	0.804 (0.70–0.88)	<0.0001
B-lines	96.4 (81.7–99.9)	82.4 (70.1–91.3)	73	97.9	0.704	0.807	0.894 (0.80–0.95)	<0.0001
Subpleural cystis	48.1 (28.7–68.1)	91.2 (80.7–97.1)	72.2	77.6	0.335	0.708	0.697 (0.58–0.79)	0.0002
Pleural effusion	14.2 (4–32.7)	96.4 (87.9–99.6)	66.7	69.6	0.095	0.672	0.554 (0.44–0.66)	0.132

CT: computed tomography; CUI: clinical utility index; GGO: ground glass; ILD: interstitial lung disease; transthoracic LUS: lung ultrasound; NSIP: non-specific interstitial pneumonia; NPV: negative predictive value; PPV: positive predictive value; UIP: usual interstitial pneumonia.

**Table 3:** Comparison between progressive ILD and non-progressive ILD of the main demographic and instrumental characteristics.

	Progressive ILD	Non-Progressive ILD	*p*-Value
*n*	51	15	
F/M *n* (%)	47 (92)/4 (8)	15 (100)/0 (0)	0.347
Age (years) (m ± sd)	60.1 ± 12.4	59.9 ± 10.2	0.936
Disease duration (years) (m ± sd)	13.3 ± 5.8	9.2 ± 4.1	0.013
Presence of LUS abnormalities			
Sliding sign *n* (%)	4 (8)	0 (0)	0.279
Pleural line irregularity *n* (%)	36 (71)	6 (40)	0.033
Pleural thickness *n* (%)	19 (37)	5 (33)	0.781
B-lines *n* (%)	37 (72)	8 (53)	0.160
B-lines number (m ± sd)	75.3 ± 31.6	48.7 ± 33.7	0.207
Subpleural cystis *n* (%)	11 (21)	4 (26)	0.679
Pleural effusion *n* (%)	2 (4)	0 (0)	0.436
PFT			
FVC% (m ± sd)	87.2 ± 17.6	101.8 ± 19.7	0.008
FEV1 (m ± sd)	86.3 ± 16	91.6 ± 18.7	0.051
DLCO% (m ± sd)	61.5 ± 17.8	76.7 ± 16	0.006
TLC% (m ± sd)	80.4 ± 16.6	101.1 ± 23.7	0.004
RV% (m ± sd)	80.6 ± 22.3	116.6 ± 48.2	0.002
6MWT (meter) (m ± sd)	446.0 ± 82.3	590.8 ± 96.3	0.086
Chest HR-TC			
UIP	6 (12)	1 (7)	0.853
NSIP with GGO	20 (39)	5 (33)	0.849
NSIP with GGO and reticulation	7 (14)	2 (13)	0.592

6MWT: 6 min walking test; CT: computed tomography; DLCO: diffusing capacity of the lungs for carbon monoxide; F: female; FEV1: forced expiratory volume in the first second; FVC: forced vital capacity; GGO: ground glass; ILD: interstitial lung disease; LUS: lung ultrasound; M: male; NSIP: non-specific interstitial pneumonia; PFT: pulmonary function tests; RV: residual volume; TLC: total lung capacity; UIP: usual interstitial pneumonia.

## Data Availability

The data presented in this study are available on request from the corresponding author.

## Supplementary Materials

The following supporting information can be downloaded at https://www.mdpi.com/article/10.3390/diagnostics15040488/s1, Table S1. True positive, true negative, false positive, false negative.

## Abbreviations

The following abbreviations are used in this manuscript:

BMI	body mass index
CRP	C-reactive proteins
ESR	erythrocyte sedimentation rate
GGO	ground glass opacities
LUS	lung ultrasound
ILD	interstitial lung disease
Lung HR-CT	chest high-resolution computed tomography
NSIP	non-specific interstitial pneumonia
PFT	pulmonary function tests
ROC	receiver operating characteristic
SSc	systemic sclerosis
UIP	usual interstitial pneumonia
