# Supplementary material for: Transthoracic Lung Ultrasound in Systemic Sclerosis-Associated Interstitial Lung Disease: Capacity to Differentiate Chest Computed-Tomographic Characteristic Patterns
Source: Diagnostics (Basel). 2025 Feb 17;15(4):488. doi: 10.3390/diagnostics15040488 (PMC11854868; doi:10.3390/diagnostics15040488)
Supplement: Supplementary file 1 [file diagnostics-15-00488-s001.zip › diagnostics-3428617-supplementary.pdf]

Supplementary Table S1. True positive, true negative, false positive, false negative.

	TP	FP	TN	FN
**UIP**				
Sliding sign	5	0	57	20
Pleural line irregularity	24	15	42	1
Pleural thickness	18	2	55	7
B-lines	14	10	47	11
Subpleural cystis	14	5	52	11
Pleural effusion	4	2	55	21
**NSIP with GGO**				
Sliding sign	2	0	57	44
Pleural line irregularity	37	15	42	9
Pleural thickness	16	2	55	30
B-lines	43	10	47	3
Subpleural cystis	10	5	52	36
Pleural effusion	2	2	55	44
**NSIP with GGO and reticulation**				
Sliding sign	5	0	57	23
Pleural line irregularity	27	15	42	1
Pleural thickness	18	2	55	10
B-lines	27	10	47	1
Subpleural cystis	13	5	52	15
Pleural effusion	4	2	55	24

FN: false negative; FP: false positive; NSIP: non-specific interstitial pneumonia; TN: true negative; TP: true positive; UIP: usual interstitial pneumonia
